# Analysis of Efficient Spectrum Handoff in a Multi-Class Hybrid Spectrum Access Cognitive Radio Network Using Markov Modelling

**DOI:** 10.3390/s19194120

**Published:** 2019-09-23

**Authors:** Atif Shakeel, Riaz Hussain, Adeel Iqbal, Irfan Latif Khan, Qadeer ul Hasan, Shahzad Ali Malik

**Affiliations:** Department of Electrical and Computer Engineering, COMSATS University Islamabad (CUI), Islamabad 45550, Pakistan; rhussain@comsats.edu.pk (R.H.); adeeliqbal@comsats.edu.pk (A.I.); irfan_latif@comsats.edu.pk (I.L.K.); qadeer.hasan@comsats.edu.pk (Q.u.H.); smalik@comsats.edu.pk (S.A.M.)

**Keywords:** cognitive radio, continuous time Markov chain, delay-sensitive, extended data delivery time, hybrid interweave-underlay spectrum access, quality of service, spectrum handoff

## Abstract

Cognitive radio networks (CRNs) rely on sensing of the licensed spectrum of a primary network to dynamically ascertain underutilized portion of the spectrum, thus affording additional communication opportunities. In a CRN, a single homogeneous spectrum access, such as interweave only deprives the secondary users (SUs) of channel access during handoff, particularly at high primary network traffic. Therefore, providing quality-of-service (QoS) to multi-class SUs with diverse delay requirements during handoff becomes a challenging task. In this paper, we have evolved a Markov-based analytical model to ascertain the gain in non-switching spectrum handoff scheme for multi-class SUs employing hybrid interweave-underlay spectrum access strategy. To satisfy the QoS requirements of the delay-sensitive traffic, we have analyzed the impact of hybrid spectrum access scheme for prioritized multi-class SUs traffic. The results show substantial improvement in spectrum utilization, average system throughput and extended data delivery time compared to conventional CRN using interweave only spectrum access. This demonstrates the suitability of the proposed scheme towards meeting QoS requirements of the delay-sensitive SU traffic while improving the overall performance for delay-tolerant SU traffic as well.

## 1. Introduction

Cognitive radio (CR) is a promising technology, which enables opportunistic communication through sensing of the licensed spectrum of the primary network by the secondary users (SUs). By allowing the SUs opportunistic access to the underutilized portion of the licensed spectrum, the utilization of limited spectrum resources can be significantly enhanced [1,2]. Spectrum handoff provides a mean of service continuity to the SUs, interrupted by the primary users (PUs) and guarantees QoS to PUs as ceasing the ongoing secondary transmission on arrival of PU provides sufficient interference protection to PUs [3]. In general, according to channel switching policy during handoff, spectrum handoff in CRN can be categorised into (i) always-staying policy, where interrupted SU stays on the occupied channel and waits for the PU to vacate the channel to resume its transmission thus also known as non-switching spectrum handoff, and (ii) always-changing policy, where interrupted SU switches to another suitable target channel and resumes its unfinished transmission [4,5].

Due to high fluctuation in the available spectrum in CRN, SUs may be interrupted multiple times before service completion, forcing them to perform multiple spectrum handoff leading to increased transmission delays. Reducing transmission delay of SUs is one of the prime objectives of an efficient spectrum handoff scheme [6]. According to delay requirements of various wireless applications, SU traffic can be classified into two main types: (i) delay-sensitive traffic, originating from real time applications e.g., voice over internet protocol (VoIP) and (ii) delay-tolerant traffic, originating from data applications e.g., a large file transfer [7,8,9]. Providing QoS to the multi-class SUs while ensuring the sufficient protection to the PUs from the interference is a very challenging task. Spectrum access strategy in CRN plays important role in avoiding the interference caused to the PUs as well as determining the performance of SUs through transmission power control [10].

In CRN, spectrum access strategies are classified mainly into three approaches [11], i.e., (i) interweave, (ii) underlay and (iii) overlay. In interweave spectrum access, SUs detect the presence of PU and access the spectrum only when it is not in use by the PU. When PU reclaims the channel the SU ceases its transmission and performs spectrum handoff either according to always-staying policy or always-changing policy. In underlay spectrum access PU and SU coexist in a spectrum simultaneously in such a way that the interference caused by the SU transmission remains within tolerable limits as imposed by the PU. To achieve this an SU has to transmit at reduced power, which limits the maximum achievable data rate of SU traffic but at least maintains the connectivity [12]. Similar, to underlay spectrum access, an overlay access strategy allows both the PU and SU to exploit the spectrum simultaneously with SU transmitting with full power, however, it requires advanced encoding techniques to be used by SUs [13].

Conventional CRN uses single homogenous spectrum access strategy, such as interweave only, which does not fully exploit the scarce spectrum resources. In this paper, we have evolved a Markov-based analytical model to evaluate the performance of non-switching spectrum handoff scheme for a CRN employing hybrid interweave-underlay spectrum access for prioritized multi-class SU traffic with focus on providing QoS to the delay-sensitive SUs.

### Contributions

The contributions of this paper are listed as below:

- The behaviour of primary user activities and its impact on multi-class SUs in hybrid CRN is thoroughly captured through (continuous time Markov chain) CTMC modelling.
- Steady state analysis is performed to analyze spectrum utilization, throughput and extended data delivery time (EDDT) of the system.
- We consider power constrained variable service rate for prioritized multi-class SUs operating in hybrid access CRN.
- A performance comparison of non-switching spectrum handoff for a multi-class SU using hybrid interweave-underlay spectrum access is made with the spectrum handoff in conventional CRN.
- To the best of our knowledge the improvement in extended data delivery time of the delay-sensitive SUs is analyzed by utilizing the steady state Markov analysis in a hybrid CRN with prioritized multi-class SUs with due consideration of power constrained transmission rate for the first time in literature.

The above mention contributions complements some of the recently published related work addressing spectrum handoff management for prioritized SU traffic and hybrid interweave-underlay spectrum access in cognitive radio networks.

## 2. Related Work

The prioritized SUs traffic in CRNs requiring spectrum handoff has been thoroughly studied in [14,15,16,17]. A non-preemptive queuing policy is presented for prioritized SU data traffic in Ref. [14] in which SU traffic is categorized into urgent, realtime and non-realtime, with urgent traffic have the highest priority and non-realtime have the lowest. Due to non-preemptive queuing policy considered for the SU traffic non-realtime packets are deprived of channel access until all the highest priority traffic finish their transmissions. This scheme ensures the QoS to the priority SUs but deprives the low priority SUs for long durations due to forced termination by the priority traffic. Channel bonding mechanism is used to combine successive time slots to transmit large data packets. The performance of secondary network is greatly affected, particularly in a high primary network environment. The impact of primary network traffic is not well studied in this work. Authors in [15] proposed a mixed preemptive resume priority—non-preemptive resume priority (PRP−NPRP) queuing model to characterize the spectrum usage behaviour of multi-class SUs. The service queuing discipline between PU and SU is preemptive, whereas within the secondary network the service queuing policy is non-preemptive, i.e., SU is allowed to complete its transmission regardless of its priority and can only be preempted by the PU. This reduces the number of handoffs for low priority users, but at the cost of higher delays for high priority delay-sensitive users. The authors analyzed the impact of PU traffic load on the transmission delay and presented a comparison for switching and non-switching spectrum handoff cases. The scheme works better when the arrival rate of SU is less otherwise the average transmission delay increases with the increase of SU traffic load. In [16] authors presented a PRP queuing policy to effectively manage the spectrum usage by new entrant SUs and the interrupted SUs; the priority is given to the interrupted SUs over new entrants. This work reduces the average service time of the SUs and also improves the QoS of the interrupted SUs. In [17] traffic adaptive spectrum handoff is proposed for multi-class SUs to minimize the handoff delay. Authors use PRP queuing to characterize multiple interruptions under different handoff strategies, considering diverse priorities for multi-class SUs. Existing work, such as [14,15,16,17], for prioritized SU traffic does not consider PU traffic intensity during spectrum handoff. However, this is an important consideration particularly during high primary traffic intensity as interrupted SUs during the handoff may suffer a call drop due to unavailability of opportunity for an extended period. In addition these work consider an interweave only spectrum access strategy for analyzing the problem of prioritized SU traffic during spectrum handoff. The interweave only and underlay only spectrum access strategies individually are unable to exploit the spectrum most effectively.

Recently, hybrid interweave-underlay spectrum access strategy is being used to improve throughput of the system for a uni-class, non-prioritized SU traffic [18,19,20,21,22]. The initial efforts towards hybrid spectrum access only consider either uni-class SUs without consideration of power constrained data rate or do not considered spectrum handoff performance metrics. Authors in [18] analyze the impact of hybrid spectrum access on the overall throughput of the system. The throughput of the system is improved by using the hybrid spectrum access, however, the study is limited to enhancing throughput of a network considering uni-class SU without evaluating the spectrum handoff performance in terms of transmission delay. Authors in [19] proposed a hybrid overlay-underlay spectrum access to improve the throughput and outage probability of the SUs. The power constrained data rate in hybrid CRN is well investigated and compared with conventional underlay only spectrum access. In [20,21] authors studied hybrid interweave-underlay access, which integrates relaying mechanism in a cooperative CRN. A Markov chain analysis is used to derive steady state probabilities of spectrum access. However, the focus of the authors is to evaluate the performance in terms of outage probability and capacity in a cooperative CRN integrating amplify and forward relaying. Similarly in [22] authors presented a queuing mechanism for analyzing the impact of primary user delay tolerance on the performance of multi-class SUs in a hybrid interweave-underlay CRN. The focus is to maximize the average service rate and minimizes the average delay of multi-class SUs. Authors in [23,24] proposed an opportunistic access scheme for a distributed CRN wherein concurrent coexistence of SUs along with primary network relies on capturing the power control messages of the primary network and then adjusting their transmission parameters in such a way that the signal to interference plus noise ratio (SINR) at the primary receiver remains above the threshold.

The Markov modelling and analysis of spectrum handoff under hybrid interweave-underlay access is very limited that does not evaluate performance metrics such as steady state probabilities, spectrum utilization, throughput and extended data delivery time for prioritized SUs with due consideration of power constrained data rate. Authors in [25] proposed a primary prioritized Markov approach for spectrum access in a conventional CRN having uni-class SU traffic. The power constrained data rate and its impact on the service rate is not taken into consideration. Authors in [26] proposed a Markov model using hybrid interweave-underlay spectrum access under delay constraint to improve the spectrum utilization for uni-class SU traffic while keeping the interference temperature within bounds. Authors in [27] proposed a Markov model for adaptive spectrum handoff in a uni-class SU traffic. The transmission delay of the interrupted SUs has been improved by effectively switching between switching and non-switching spectrum handoff. Authors in [28] presented a steady state analysis for modelling the behaviour of primary and secondary users in a uni-class primary prioritized conventional CRN. Similarly, authors in [29] proposed a Markov model with channel reservation to improve the spectrum utilization. The performance of the interrupted SUs is improved with reserved channel however, the delay characteristics of the SUs is not well studied. The work in [30] analyzed the performance of prioritized multi-class SUs using continuous time Markov chain for centralized and distributed DSA schemes. The QoS requirements of the high priority SUs was achieved by reservation of sub channel during the spectrum handoff process. However, this work did not consider hybrid spectrum access. Authors in [31] proposed a hidden Markov model (HMM) with channel state prediction for dynamic spectrum access in a CRN. The sensing delay in a cognitive cycle was improved by skipping the channels predicted busy in the sensing phase, thus only sensing the channels predicted idle. The Markov decision process (MDP) in this work has been applied for the improvement of spectrum sensing, however, its effect on spectrum handoff process remains to be investigated.

Table 1 presents the comparison of related work and the proposed in terms of aspect considered. The categorization of existing work is done using the main idea of work, use of hybrid spectrum access and Markov model, prioritized multi-class traffic, consideration of power constrained data rate in hybrid CRN, spectrum utilization and transmission delay. Our work encompasses all of these aspects.

The work in this paper differs from the earlier research in such a way that we have considered hybrid spectrum access for providing QoS to multi-class SUs having diverse priorities. Unlike existing Markov-based analysis in hybrid interweave-underlay access, our approach takes into consideration varying rates of departure for multi-class SUs operating in interweave and underlay access.

The rest of the paper is organized as follows: Section 3 describes the system model for hybrid interweave-underlay spectrum access for multi-class SUs. Section 4 presents the CTMC modelling and steady state analysis to study the interaction between PUs and multi-class SUs. Steady state probabilities are analyzed for different critical scenarios. Section 5 provides results and finally, conclusion is given in Section 6.

## 3. System Model

**Figure 1:**
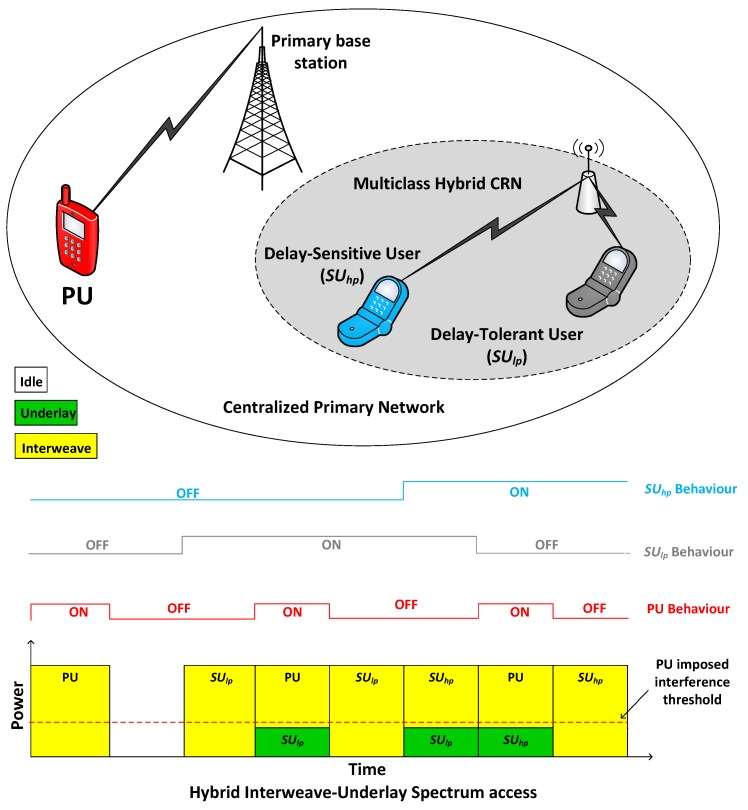
System Model of Multi-class Hybrid Interweave-Underlay CRN.

We consider a centralized CRN with a PU and two classes of SUs with traffic classified based on their service delay requirements, i.e., (i) SUs with delay-sensitive traffic, coming from a real time applications having a higher priority and (ii) SUs with delay-tolerant traffic, coming from general application, e.g., large file transfer, having low priority in accessing an idle channel. We denote high priority delay-sensitive user as $SU_{hp}$, low priority delay-tolerant user as $SU_{lp}$ and primary user as PU, as shown in the Figure 1.

The traffic arrival of PU is considered as an independent Poisson process and the inter-arrival time is exponentially distributed. The service queuing discipline between primary and secondary network users is considered as a (Preemptive Resume Priority) PRP, where PU can preempt the SUs of any class during their service. Similarly, within the secondary network, $SU_{hp}$ can preempt user $SU_{lp}$ in order to meet its QoS requirements. The coordination among multi-class SUs during spectrum access is provided by the centralized entity. We considered non-switching spectrum handoff policy in which the interrupted SUs has to stay on the current channel and wait for the PU to vacate the channel to resume its undone transmission [32].

We assume a hybrid interweave-underlay spectrum access in CRN, which exploits the benefits of both interweave and underlay spectrum access techniques under power constrain [33]. Therefore, the maximum achievable data rate (r) of user γ where $\gamma \in \left\{PU,SU_{hp},SU_{lp}\right\}$ when operating alone in the channel in the case of interweave spectrum access mode is given by [34]:

(1) $$r=W\log_{2}\left(1+\frac{P_{\gamma }G_{\gamma }}{N}\right),$$

where, *W* is the communication bandwidth, $P_{\gamma }$ is the transmission power of the user γ, $G_{\gamma }$ is the channel gain for user γ, and *N* is the power of additive white Gaussian noise (AWGN). In underlay access, the SU has to reduce its transmit power in order to keep the interference caused to PU below the threshold, which reduces the maximum data rate $r_{u}^{\gamma }$ of the SU operating in underlay access as given by:

(2) $$r_{u}^{\gamma }=W\log_{2}\left(1+\frac{P_{\gamma }G_{\gamma }}{N+P_{p}G_{p\gamma }}\right),$$

where, $G_{p\gamma }$ is the channel gain from PU transmitter to γ receiver, $P_{p}$ is the transmission power of the PU and $P_{\gamma }$ is the transmission power of the user γ operating in underlay access mode. The behaviour of multi-class SU in hybrid CRN is shown in Figure 1, where $SU_{hp}$ or $SU_{lp}$ switches between interweave and underlay spectrum access strategy. The arriving SU from either class first senses the spectrum for the presence of PU before transmission. Once the channel is found idle, SU transmits in the rest of the time frame using interweave spectrum access, otherwise it goes to underlay spectrum access mode and transmit concurrently with the PU. The idle and busy state of the channel relies on the binary hypothesis $H_{0}$ and $H_{1}$ for the received signal x(t), where $H_{0}$ represents the idle state and $H_{1}$ represent the busy state of the channel [35,36].

(3) $$x\left(t\right)=\left\{\begin{array}{cc} n\left(t\right) & H_{0}\\ p\left(t\right)+n\left(t\right) & H_{1}, \end{array}\right.$$

Here, x(t) is the received signal, n(t) is the AWGN signal and p(t) is the transmitted signal of the PU. We assume perfect sensing results and ignore the spectrum sensing errors, i.e., miss detection and false alarm.

## 4. Continuous Time Markov Chain Modelling and Steady State Analysis

**Figure 2:**
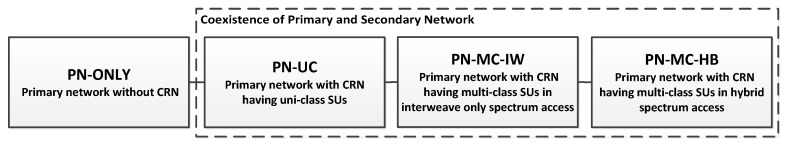
Set of cases for continuous time Markov chain (CTMC) Modelling.

In this section, we present CTMC modelling for set of critical cases as shown in Figure 2 to study the dynamics of PU and SU and subsequently evolve the analytical model for hybrid spectrum access with multi-class SUs. We start with the study of a primary network without a CRN. In the second step, we consider a primary network with CRN having uni-class SUs. Thirdly, we consider a CRN with multi-class SUs using interweave spectrum access strategy and study the effect of primary traffic intensity on the transmission delay and throughput of multi-class SUs. Finally, we consider a CRN with multi-class SUs using hybrid interweave-underlay spectrum access to meet the QoS requirements of multi-class SUs. Steady state analysis for all cases are performed, which is utilized for performance evaluation.

### 4.1. Primary Network without CRN (PN-ONLY)

**Figure 3:**
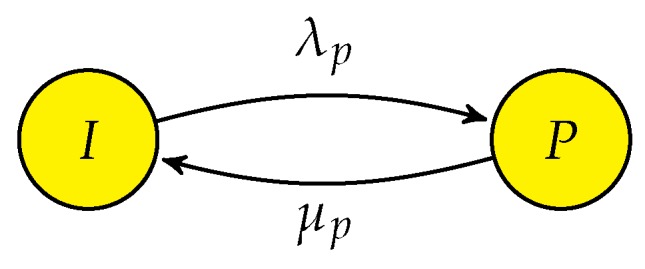
State Transition Diagram of PN-ONLY.

The state transition diagram for the primary network without CRN is shown in Figure 3. The spectrum is only utilized by the PUs. CTMC model has only two states in this case. State *I* and state *P*, where state *I* represents idle state and state *P* represents that PU is using the spectrum. The traffic arrival of PU is considers as a Poisson process with rate $\lambda_{p}$ and the corresponding service time is exponentially distributed with rate $\mu_{p}$. Assume that the system is initially in state *I* and on arrival of PU, system transits into to state *P* with rate of transition $\lambda_{p}$. After completing its service, PU vacates the channel and the system returns to state *I* with rate $\mu_{p}$. The state space vector of the system is given as $S=\left\{I,P\right\}$. We denote this CTMC as “PN-ONLY”.

The transition rate matrix *Q* of the PN-ONLY is given in (4), which shows the rate of transition between different states of vector *S*.

(4) $$Q=\begin{array}{cc} & \begin{array}{cc} \;Idle & \;P \end{array}\\ \begin{array}{c} Idle\\ P \end{array} & \left[\begin{array}{cc} -\lambda_{P} & \lambda_{P}\\ \mu_{P} & -\mu_{P} \end{array}\right] \end{array}$$

The flow balance equation [37], which is the rate at which transition takes out of state β∈S becomes equal to the rate of transition into state β∈S for PN-ONLY is given by:

(5) $$\left.\begin{array}{c} \pi_{i}\lambda_{p}=\pi_{p}\mu_{p}, \end{array}\right.$$

in addition, the normalization condition is given as:

(6) $$\sum_{\beta \in S}\pi_{\beta }=1,$$

the steady state probabilities $\pi_{i}$ and $\pi_{p}$ obtained by solving (5) and (6) in this case are as follows:

(7) $$\pi_{i}=\frac{\mu_{p}}{\lambda_{p}+\mu_{p}},$$

(8) $$\pi_{p}=\frac{\lambda_{p}}{\lambda_{p}+\mu_{p}}.$$

**Figure 4:**
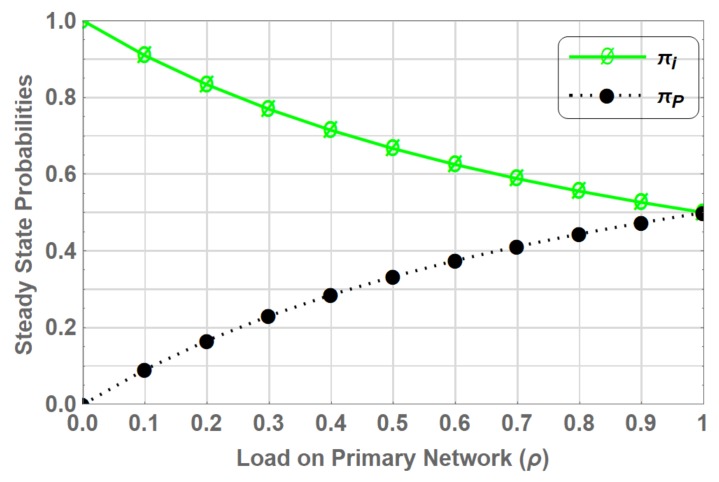
Stationary probabilities for PN-ONLY.

Figure 4 shows the steady state probabilities $\pi_{\beta }$ where β∈S for varying load on primary network (ρ). The graph shows that even at peak primary traffic load there is room for the secondary network to opportunistically access the underutilized portion of the spectrum.

### 4.2. Primary Network with CRN Having Uni-Class SUs (PN-UC)

**Figure 5:**
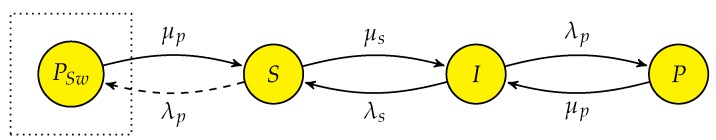
State Transition Diagram of CTMC PN-UC, “--->” indicates spectrum handoff event.

The PN-ONLY is extended with the inclusion of secondary network in which SUs opportunistically access the unused spectrum without causing interference to the PU. The SU traffic considered in this case is uni-class. The traffic arrival of both PUs and SUs is considered as an independent Poisson process with rate $\lambda_{p}$ and $\lambda_{s}$ respectively, and the service time is considered as exponentially distributed with rate of departure $\mu_{p}$ and $\mu_{s}$, respectively. The state transition diagram is shown in Figure 5, where state *I* represents that there is neither PU nor SU employing the spectrum, state *P* represents PU is using the spectrum, state *S* represents that SU is using the spectrum and state $P_{Sw}$ represents that PU is using the spectrum while interrupted SU is waiting on the channel to become idle again.

Assume that the system is in state *I* at the beginning. The system transits to either state *P* or state *S* on the arrival of PU or SU with rate $\lambda_{p}$ or $\lambda_{s}$, respectively. From their respective states, both PU and SU returns to state *I* with rate of departure $\mu_{p}$ and $\mu_{s}$, respectively, on completing their transmission. As a licenced user, PU may preempt the SU anytime during the transmission, therefore forcing the SU to perform spectrum handoff. The transition from state *S* to state $P_{S_{w}}$ take place when SU is preempted by the PU with rate of arrival $\lambda_{p}$, this event reflects the non- switching spectrum handoff process, where PU occupy the channel and SU goes to waiting state, as shown by the dashed line in Figure 5. When the PU departs with rate $\mu_{p}$, SU resumes its transmission on the channel and state changes from $P_{S_{w}}$ to state *S*. The state space vector of the system in this case is $S=\left\{I,P,S,P_{Sw}\right\}$ and the transition rate matrix *Q* is given in (9). We denote this CTMC as “PN-UC”.

(9) $$Q=\begin{array}{cc} & \begin{array}{cccc} I\;\;\; & \;P\;\; & \;S\; & P_{Sw} \end{array}\\ \begin{array}{c} I\\ P\\ S\\ P_{Sw} \end{array} & \left[\begin{array}{rrrr} -(\lambda_{p}+\lambda_{s}) & \lambda_{p} & \lambda_{s} & 0\\ \mu_{p} & -\mu_{p} & 0 & 0\\ \mu_{s} & 0 & -(\lambda_{p}+\mu_{s}) & \lambda_{p}\\ 0 & 0 & \mu_{p} & -\mu_{p} \end{array}\right] \end{array}$$

The flow balance equations of PN-UC are given by:

(10) $$\left.\begin{array}{c} \pi_{i}\left(\lambda_{p}+\lambda_{s}\right)=\pi_{p}\mu_{p}+\pi_{s}\mu_{s},\\ \pi_{p}\mu_{p}=\pi_{i}\lambda_{p},\\ \pi_{s}\left(\lambda_{p}+\mu_{s}\right)=\pi_{i}\lambda_{s}+\pi_{psw}\mu_{p},\\ \pi_{p_{sw}}\mu_{p}=\pi_{s}\lambda_{p}, \end{array}\right.$$

solving the set of linear equations in (10) together with the normalization condition given as:

(11) $$\sum_{\beta \in S}\pi_{\beta }=1,$$

the steady state probabilities $\pi_{i},\pi_{p}$, $\pi_{s}$ and $\pi_{p_{sw}}$ are as follows:

(12) $$\pi_{i}=\frac{\mu_{p}\mu_{s}}{\left(\lambda_{p}+\mu_{p}\right)\left(\lambda_{s}+\mu_{s}\right)},$$

(13) $$\pi_{p}=\frac{\lambda_{p}\mu_{s}}{\left(\lambda_{p}+\mu_{p}\right)\left(\lambda_{s}+\mu_{s}\right)},$$

(14) $$\pi_{s}=\frac{\lambda_{s}\mu_{p}}{\left(\lambda_{p}+\mu_{p}\right)\left(\lambda_{s}+\mu_{s}\right)},$$

(15) $$\pi_{p_{sw}}=\frac{\lambda_{p}\lambda_{s}}{\left(\lambda_{p}+\mu_{p}\right)\left(\lambda_{s}+\mu_{s}\right)}.$$

**Figure 6:**
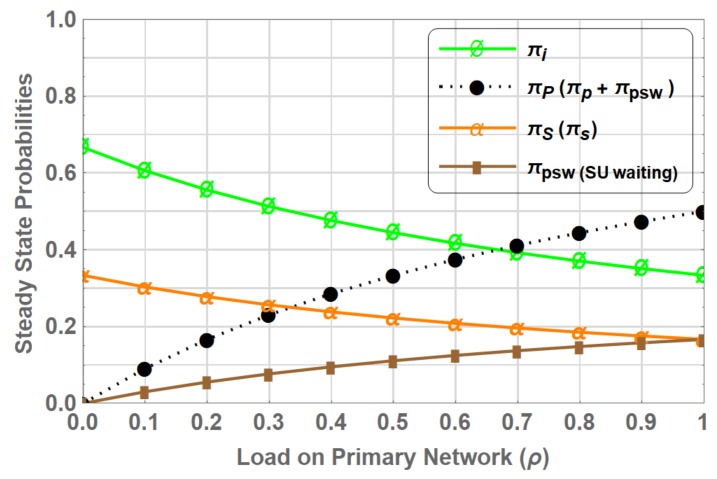
Stationary probabilities for PN-UC.

Figure 6 shows the steady state probabilities of PN-UC for varying ρ. $\pi_{P}$ is the steady state probability showing the presence of PU in the system, which is sum of $\pi_{p}$ and $\pi_{psw}$, as PU is occupying the channel in both states *P* and $P_{sw}$. Whereas $\pi_{S}$ is the steady state probability representing the presence of SU in the system, which is only reflected when the system is in state *S* which has steady state probability of $\pi_{s}$. Compared to PN-ONLY the $\pi_{P}$ remains exactly the same, which indicates that the presence of CRN does not affect the primary network as SUs access the spectrum opportunistically. In fact due to the presence of secondary network the portion of time the spectrum is idle, i.e., $\pi_{i}$ decreases as the spectrum is being utilized by the SUs in the absence of PUs, eventually improves the spectrum utilization.

It is also observed that as the ρ increases the number of opportunities for SU decreases as reflected by the decreasing trend of $\pi_{S}$ and increasing trend of $\pi_{p_{sw}}$. At ρ=1, $\pi_{S}$ is at its minimum due to the limited number of opportunities available and $\pi_{psw}$ is at its maximum due to frequent interruption caused by the high PU reappearance probability.

### 4.3. Primary Network with CRN Having Multi-Class SUs in Interweave only Spectrum Access (PN-MC-IW)

**Figure 7:**
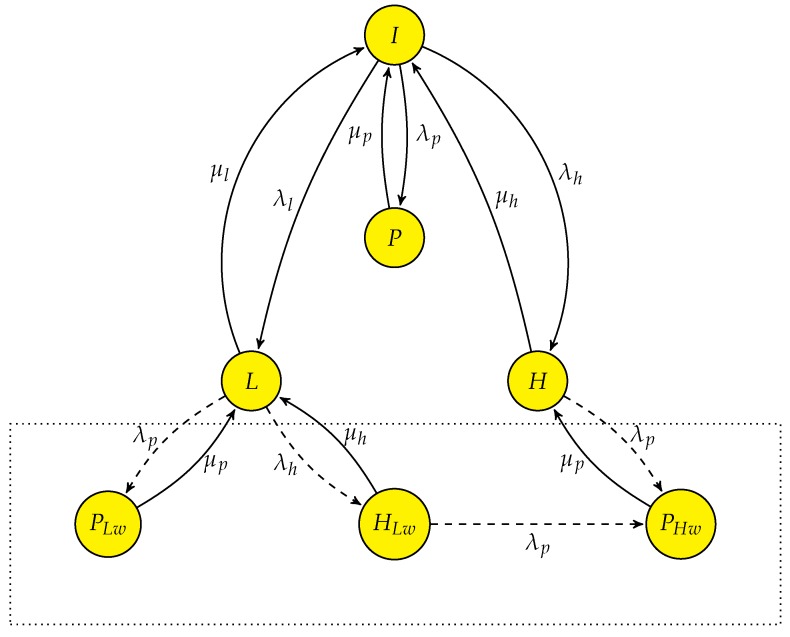
State transition diagram of PN-MC-IW, “--->” indicates spectrum handoff event.

The state transition diagram for multi-class SUs in a conventional CRN using interweave only spectrum access is given in Figure 7. Based on the delay requirements, there is a high priority delay-sensitive user $\left(SU_{hp}\right)$ and low priority delay-tolerant user $\left(SU_{lp}\right)$. The spectrum access process is modelled as a seven state CTMC where state *I* means system is in idle state, state *P* means PU is operating in the system, state *H* and *L* means $SU_{hp}$ and $SU_{lp}$ operating in the system, respectively. State $P_{Hw}$ and $P_{Lw}$ represent that PU is using the spectrum while $SU_{hp}$ and $SU_{lp}$ are waiting respectively. Similarly state $H_{Lw}$ represents $SU_{hp}$ is using the spectrum and $SU_{lp}$ is waiting. It is assumed that at any given time only a single SU can be in waiting state when PU occupies the channel. Assume system is in state *I* in the beginning, the arrival of PU transits the system to state *P* with rate $\lambda_{p}$. On completing its transmission PU departs with rate $\mu_{p}$ and system returns to state *I*. As in interweave spectrum access, SUs are only able to access the channel when PU is not present. Therefore, system transfers from state *I* to *H* or *L* on the arrival of $SU_{hp}$ or $SU_{lp}$ with Poisson arrival $\lambda_{h}$ or $\lambda_{l}$, respectively. When $SU_{hp}$ or $SU_{lp}$ completes its transmission, the system returns to state *I* from the respective state with exponentially distributed departure time and rate of $\mu_{h}$ or $\mu_{l}$, respectively. Collectively $\mu_{p}$ and $\mu_{l}$ would be slow decaying heavy-tailed. However, as we have considered *L* and $P_{Lw}$ as two separate states the state transition from state $P_{Lw}$ to *L* and from *L* to *I* are Poisson distributed. PU being the licensed user can preempt both $SU_{hp}$ and $SU_{lp}$ during their transmission forcing them to perform spectrum handoff. So, interrupted SU has to cease its transmission and wait for the channel to become idle again.

State $P_{Lw}$ is achieved when a $SU_{lp}$ is interrupted by a PU, which forces it to perform a non-switching handoff. Once PU departs with rate $\mu_{p}$ the $SU_{lp}$ becomes active and resumes its transmission on the same channel and departs with rate $\mu_{l}$. Similarly when $SU_{hp}$ is preempted by PU, state transits from *H* to $P_{Hw}$.

To meet QoS requirements of delay-sensitive user, $SU_{hp}$ can preempt $SU_{lp}$ during its transmission, so $SU_{lp}$ can only access the channel when there is neither PU nor $SU_{hp}$ in the system. There are four handoff events in this case as represented by the dashed line in the Figure 7, i.e., (i) when PU preempted the $SU_{hp}$, state *H* transfers into state $P_{Hw}$, (ii) when $SU_{lp}$ is preempted by the PU during its service CTMC transfers from state *L* to state $P_{Lw}$, (iii) when $SU_{hp}$ preempted $SU_{lp}$ state transfers from *L* to $H_{Lw}$ and iv) when system transits from state $H_{Lw}$ to $P_{Hw}$ when $SU_{hp}$ is interrupted by the PU. $SU_{hp}$ having the higher priority then $SU_{lp}$ goes into waiting and $SU_{lp}$ is dropped from the system. The state space vector of the system in this case is $S=\left\{I,P,H,L,P_{Hw},P_{Lw},H_{Lw}\right\}$ and the transition rate matrix *Q* is given as in (16). We denote this CTMC as “PN-MC-IW”.

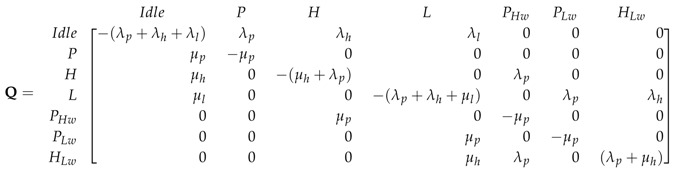
(16)

The flow balance equations governing the above system are given by:

(17) $$\left.\begin{array}{c} \pi_{i}\left(\lambda_{p}+\lambda_{l}+\lambda_{h}\right)=\pi_{p}\mu_{p}+\pi_{h}\mu_{h}+\pi_{l}\mu_{l},\\ \pi_{p}\mu_{p}=\pi_{i}\lambda_{p},\\ \pi_{h}\left(\lambda_{p}+\mu_{h}\right)=\pi_{i}\lambda_{h}+\pi_{phw}\mu_{p},\\ \pi_{l}\left(\lambda_{p}+\mu_{l}+\lambda_{h}\right)=\pi_{i}\lambda_{l}+\pi_{plw}\mu_{p}+\pi_{hlw}\mu_{h},\\ \pi_{phw}\mu_{p}=\pi_{h}\lambda_{p}+\pi_{hlw}\lambda_{p},\\ \pi_{plw}\mu_{p}=\pi_{l}\lambda_{p},\\ \pi_{hlw}\left(\lambda_{p}+\mu_{h}\right)=\pi_{l}\lambda_{h}, \end{array}\right.$$

and the normalization equation that satisfies the CTMC is

(18) $$\sum_{\beta \in S}\pi_{\beta }=1.$$

The steady state probabilities for each state $\pi_{\beta }$ can be found by solving the set of linear equations in (17) along with (18) and are given as follows.

(19) $$\pi_{i}=\frac{\mu_{h}\left(\lambda_{h}\lambda_{p}+\left(\lambda_{p}+\mu_{h}\right)\mu_{l}\right)\mu_{p}}{\left(\lambda_{h}+\mu_{h}\right)\left(\lambda_{h}\lambda_{p}+\left(\lambda_{p}+\mu_{h}\right)\left(\lambda_{l}+\mu_{l}\right)\right)\left(\lambda_{p}+\mu_{p}\right)},$$

(20) $$\pi_{p}=\frac{\lambda_{p}\mu_{h}\left(\lambda_{h}\lambda_{p}+\left(\lambda_{p}+\mu_{h}\right)\mu_{l}\right)}{\left(\lambda_{h}+\mu_{h}\right)\left(\lambda_{h}\lambda_{p}+\left(\lambda_{p}+\mu_{h}\right)\left(\lambda_{l}+\mu_{l}\right)\right)\left(\lambda_{p}+\mu_{p}\right)},$$

(21) $$\pi_{h}=\frac{\lambda_{h}\left(\left(\lambda_{h}+\lambda_{l}\right)\lambda_{p}+\left(\lambda_{p}+\mu_{h}\right)\mu_{l}\right)\mu_{p}}{\left(\lambda_{h}+\mu_{h}\right)\left(\lambda_{h}\lambda_{p}+\left(\lambda_{p}+\mu_{h}\right)\left(\lambda_{l}+\mu_{l}\right)\right)\left(\lambda_{p}+\mu_{p}\right)},$$

(22) $$\pi_{l}=\frac{\lambda_{l}\mu_{h}\left(\lambda_{p}+\mu_{h}\right)\mu_{p}}{\left(\lambda_{h}+\mu_{h}\right)\left(\lambda_{h}\lambda_{p}+\left(\lambda_{p}+\mu_{h}\right)\left(\lambda_{l}+\mu_{l}\right)\right)\left(\lambda_{p}+\mu_{p}\right)},$$

(23) $$\pi_{phw}=\frac{\lambda_{h}\lambda_{p}}{\left(\lambda_{h}+\mu_{h}\right)\left(\lambda_{p}+\mu_{p}\right)},$$

(24) $$\pi_{plw}=\frac{\lambda_{l}\lambda_{p}\mu_{h}\left(\lambda_{p}+\mu_{h}\right)}{\left(\lambda_{h}+\mu_{h}\right)\left(\lambda_{h}\lambda_{p}+\left(\lambda_{p}+\mu_{h}\right)\left(\lambda_{l}+\mu_{l}\right)\right)\left(\lambda_{p}+\mu_{p}\right)},$$

(25) $$\pi_{hlw}=\frac{\lambda_{h}\lambda_{l}\mu_{h}\mu_{p}}{\left(\lambda_{h}+\mu_{h}\right)\left(\lambda_{h}\lambda_{p}+\left(\lambda_{p}+\mu_{h}\right)\left(\lambda_{l}+\mu_{l}\right)\right)\left(\lambda_{p}+\mu_{p}\right)}.$$

**Figure 8:**
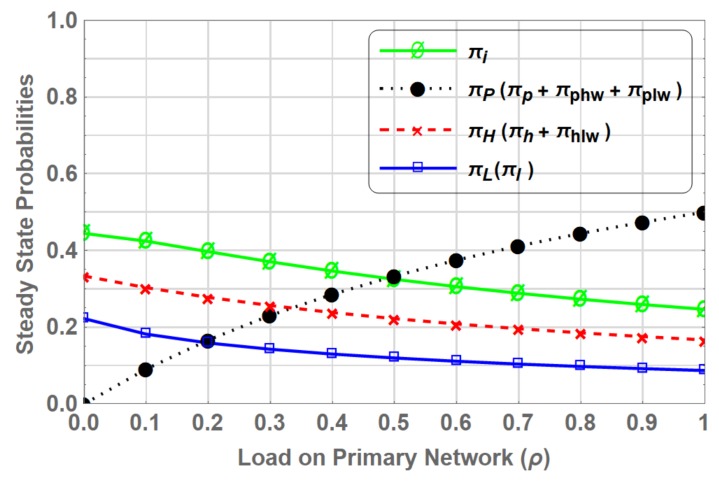
Stationary probabilities for PN-MC-IW.

Figure 8 shows the steady state probabilities $\pi_{\beta }$ where β∈S for varying values of ρ. The steady state probability $\pi_{P}$, representing the presence of PU in the system, is the sum of $\pi_{p}$, $\pi_{phw}$ and $\pi_{plw}$. $\pi_{H}$ can be obtained by adding the $\pi_{h}$ and $\pi_{hlw}$ as $SU_{hp}$ is occupying the spectrum in state *H* and $H_{Lw}$, whereas $\pi_{L}$ is only reflected by $\pi_{l}$ as $SU_{lp}$ is either in waiting state, i.e., $P_{Lw}$ and $H_{Lw}$, or not present in the system. It shows that $\pi_{H}$ is greater than the $\pi_{L}$ due to higher priority of $SU_{hp}$. At ρ=0, $\pi_{H}$ is equal to 0.333 compared to $\pi_{L}$ at 0.222 considering the SU traffic load fixed at $\lambda_{h}$ = $\lambda_{l}$ = 1 and $\mu_{h}$ = $\mu_{l}$ = 2. This dominance continues as the load on primary network increases.

### 4.4. Primary Network with CRN Having Multi-Class SUs in Hybrid Spectrum Access (PN-MC-HB)

**Figure 9:**
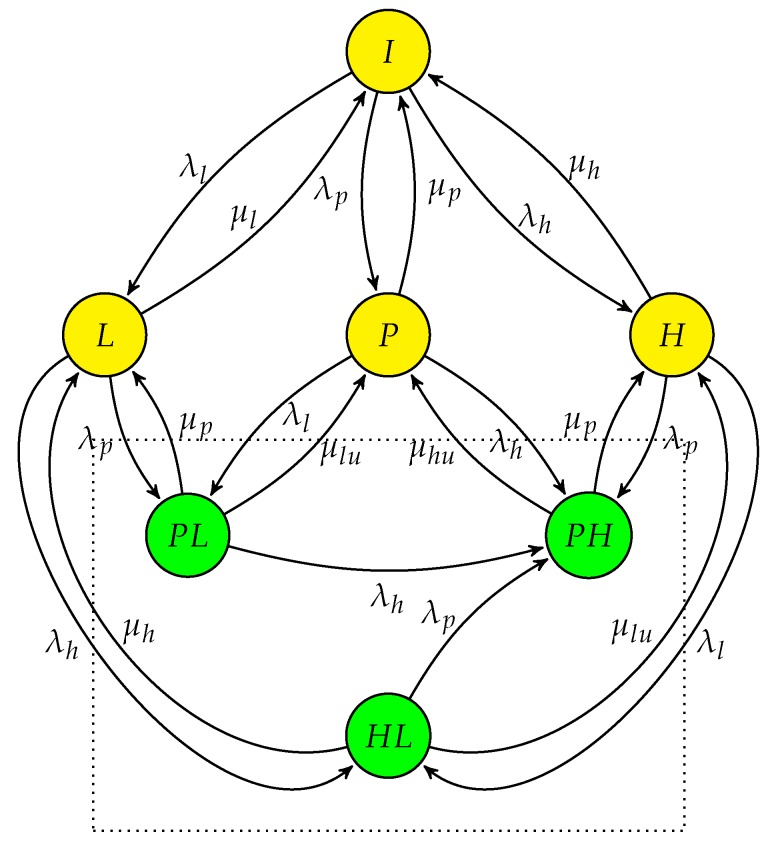
State transition diagram of PN-MC-HB.

In PN-MC-IW the interrupted SUs have to wait for long duration for channel to become free, particularly at higher primary traffic load. This eventually increases the transmission delay of the interrupted SUs and deprives the delay-sensitive SUs for achieving required QoS. The PN-MC-IW is extended in such a way that multi-class SUs when preempted by the PU, instead of going to state $P_{Hw}$ and wait for the channel to become free, instead it transits to state PH as shown in Figure 9. In state PH$SU_{hp}$ operates in an underlay access mode and rather than waiting achieves some data rate with due consideration of the interference caused to the PU.

Similarly when $SU_{lp}$ is using the spectrum and PU comes back, system transits from state *L* to state PL rather $P_{Lw}$ (as was in case of PN-MC-IW), this shows the coexistence of both PU and $SU_{lp}$. The dotted box contains three states, i.e., PH, PL and HL that represents the coexistence of PU and multi-class SUs either $SU_{hp}$ or $SU_{lp}$, operating in underlay access. We assume that only single SU can coexist with the PU operating in underlay access mode with priority is given to $SU_{hp}$ due to its delay constraint. As user operating in underlay access mode reduces its transmission power in order to keep the interference below threshold, the maximum achievable data rate is also much less as compared to interweave access. Therefore, it takes more time for a user operating in underlay access to complete its data transmission. Hence, rate of departure, $\mu_{hu}$ and $\mu_{lu}$, are much reduce in state PH and PL, where user $SU_{hp}$ or $SU_{lp}$ operates in underlay. The state space vector of the system in this case is $S=\left\{I,P,H,L,PH,PL,HL\right\}$. The transition rate matrix *Q* is given in (26).


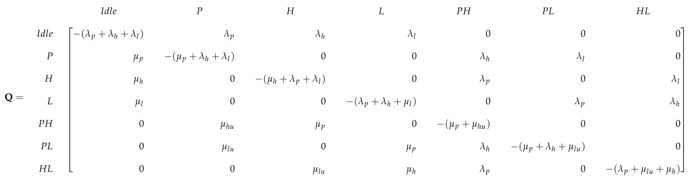
(26)

On the basis of Figure 9, the flow balance equations of PN-MC-HB are given by:

(27) $$\begin{array}{c} \pi_{i}\left(\lambda_{p}+\lambda_{l}+\lambda_{h}\right)=\pi_{p}\mu_{p}+\pi_{h}\mu_{h}+\pi_{l}\mu_{l},\\ \pi_{p}\left(\mu_{p}+\lambda_{l}+\lambda_{h}\right)=\pi_{i}\lambda_{p}+\pi_{pl}\mu_{lu}+\pi_{ph}\mu_{hu},\\ \pi_{h}\left(\lambda_{p}+\mu_{h}+\lambda_{l}\right)=\pi_{i}\lambda_{h}+\pi_{ph}\mu_{p}+\pi_{hl}\mu_{lu},\\ \pi_{l}\left(\lambda_{p}+\mu_{l}+\lambda_{h}\right)=\pi_{i}\lambda_{l}+\pi_{pl}\mu_{p}+\pi_{hl}\mu_{h},\\ \pi_{ph}\left(\mu_{p}+\mu_{hu}\right)=\pi_{h}\lambda_{p}+\pi_{p}\lambda_{h}+\pi_{pl}\lambda_{h}+\pi_{hl}\lambda_{p},\\ \pi_{pl}\left(\mu_{p}+\mu_{lu}+\lambda_{h}\right)=\pi_{l}\lambda_{p}+\pi_{p}\lambda_{l},\\ \pi_{hl}\left(\lambda_{p}+\mu_{h}+\mu_{lu}\right)=\pi_{l}\lambda_{h}+\pi_{h}\lambda_{l}, \end{array}$$

and the normalization equation that satisfies the CTMC is given as:

(28) $$\sum_{\beta \in S}\pi_{\beta }=1.$$

**Figure 10:**
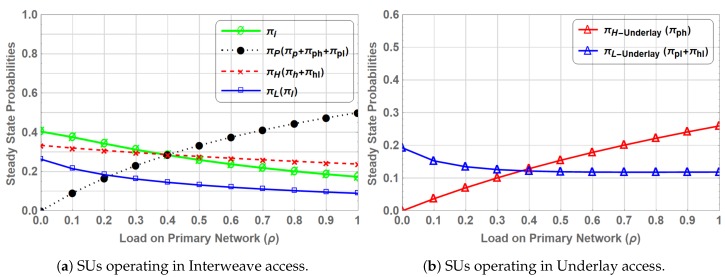
Stationary probabilities for PN-MC-HB.

The steady state probabilities for each state $\pi_{\beta }$(β∈S) can be found by solving the set of linear equations in (27) and (28). Similar to PN-MC-IW steady state probability $\pi_{P}$ is the sum of $\pi_{p}$, $\pi_{ph}$ and $\pi_{pl}$. The steady state probability $\pi_{H}$ represents when $SU_{hp}$ operates in interweave access and is the sum of $\pi_{h}$ and $\pi_{hl}$ whereas, steady state probability $\pi_{L}$ represent when $SU_{lp}$ operates in interweave access, which is only reflected by $\pi_{l}$, as $SU_{lp}$ either operates in underlay underlay access mode, i.e., states PL and HL, or is not present in the system. Figure 10a shows the steady state probabilities $\pi_{\beta }$, where β∈S for entire range of ρ, where SUs operate using interweave spectrum access in the absence of PU. The gain in PN-MC-IW is shown in Figure 10b, where SUs operate in underlay access mode. The steady state probability for $SU_{hp}$, operating in underlay access mode i.e., $\pi_{H-Underlay}$, increases as the load on primary network increases. This is due to the fact that when ρ increases SUs are preempted more frequently forcing them to operate in underlay access mode. Whereas, steady state probability for $SU_{lp}$ in underlay access mode $\left(\pi_{L-Underlay}\right)$ gradually decrease as increase in ρ. This is due to the fact that at ρ=0 user $SU_{hp}$ gets maximum opportunities to access the spectrum in the absence of PU and being the higher priority operates in interweave spectrum access while and $SU_{lp}$ operates in underlay access mode coexisting with $SU_{hp}$. However, when the ρ increases the channel is mostly occupied by the PU, which forces $SU_{hp}$ to operate mostly in underlay access mode and further reduces the $SU_{lp}$ presence in the system.

## 5. Simulation and Results

The numerical results are provided to illustrate the impact of key system parameters on the performance of the PN-MC-HB and compared with the PN-ONLY, PN-UC and PN-MC-IW schemes. Key performance parameters include steady state probabilities, spectrum utilization, system throughput, extended data delivery time. Table 2 shows the system parameters.

The load on primary network (ρ) corresponds to PU arrival and departure rate and is varied in the range [0.0–1.0]. For simplicity, we have considered a single primary channel as it is adequate to model and analyze the performance of non-switching spectrum handoff. For the PN-UC scheme, we assume the arrival rate of SU $\lambda_{s}=2$ and departure rate $\mu_{s}=4$. Similarly, for PN-MC-IW arrival of $SU_{hp}$ and $SU_{lp}$ are assumed as $\lambda_{h}=\lambda_{l}=1$, while departure rate i.e., $\mu_{h}=\mu_{l}=4$. These values have been selected such that the system stability condition, i.e., λ/μ≤1, remains intact.

As discussed in the background section the maximum achievable data rate of SU operating in interweave and underlay spectrum access are not same due to power constraint. We have considered departure rate of SU operating in underlay as 1/10th of the departure rate in interweave spectrum access, i.e., $\mu_{hu}=\mu_{lu}=0.01$. The transmission rates are as per IEEE 802.11a channel with maximum achievable transmission rate of 48 Mbps in the case of interweave and 18 Mbps in the case of underlay spectrum access strategy. The parameters considered to evaluate extended data delivery time for delay sensitive and delay tolerant SUs for transmitting a 5 Kbit file over a 22 MHz channel.

### 5.1. Steady State Probabilities

**Figure 11:**
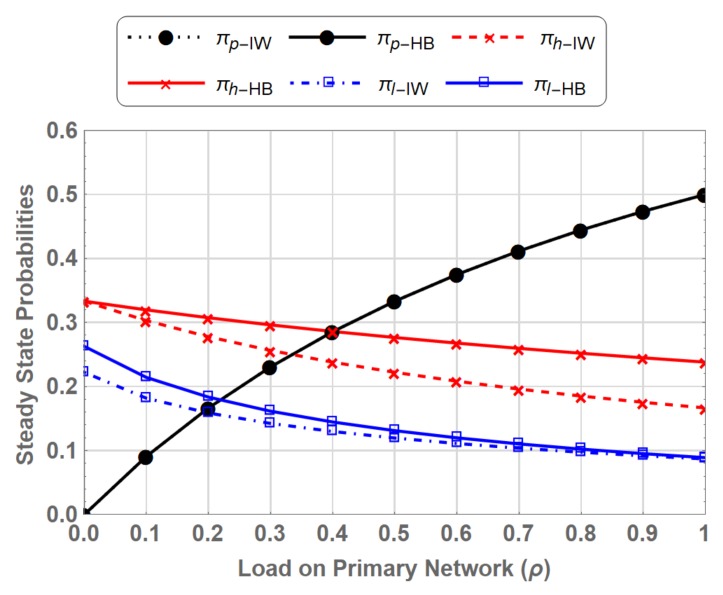
Comparison of Stationary probabilities for PN-MC-IW and PN-MC-HB.

**Figure 12:**
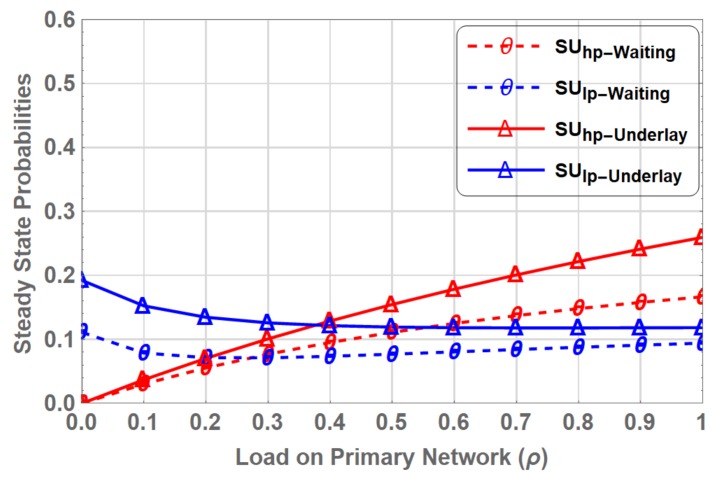
Comparison of interrupted SUs waiting vs. operating in underlay access.

Steady state probabilities represent the fraction of time the spectrum is occupied by a primary or secondary user in a particular state. Figure 11 shows comparison of steady states for PN-MC-IW and PN-MC-HB. For the considered range of ρ, the steady state probability of $\pi_{P}$ is same for both schemes. This shows that coexistence of SUs whether $SU_{hp}$ or $SU_{lp}$ along with the PU does not interfere the primary transmission. Similarly, $\pi_{H}$ and $\pi_{L}$ show identical behaviour in both cases, however, the gain in the case of PN-MC-HB over PN-MC-IW is due to the fact that $SU_{hp}$ or $SU_{lp}$ switches to underlay access mode instead of waiting on the channel when preempted by the PU. Results are shown in Figure 12.

$SU_{hp-Underlay}$ which is the portion of time $SU_{hp}$ operates in underlay mode shows an increasing trend against the ρ due to the fact that as the ρ increases the number of opportunities for secondary user whether $SU_{hp}$ or $SU_{lp}$ decrease, forcing the SU to switch to underlay access mode. $SU_{lp-Underlay}$ starts at 0.192 by keeping the arrival and departure rates of $SU_{lp}$ fixed we observe a decreasing trend of $SU_{lp-Underlay}$ as the ρ increases. This is because $SU_{hp}$ operates in underlay mode more often due to frequent preemption by PU at high load thus leaving little room for $SU_{lp}$ to coexist with PU.

### 5.2. Spectrum Utilization

**Figure 13:**
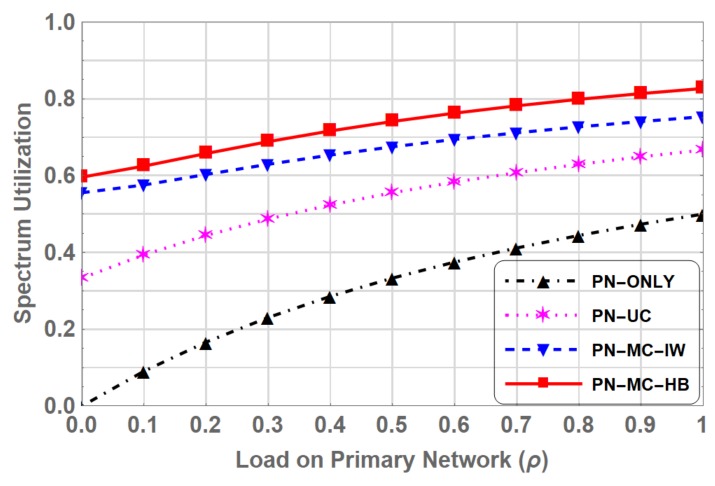
Comparison of Spectrum Utilization for PN-ONLY, PN-UC, PN-MC-IW and PN-MC-HB.

Spectrum utilization is the ratio of occupation time of the spectrum either by PUs or SUs to the total time [38]. Spectrum utilization increases as the number of users occupying the spectrum increases. Comparison of spectrum utilization between PN-ONLY, PN-UC, PN-MC-IW and PN-MC-HB is shown in Figure 13.

**Figure 14:**
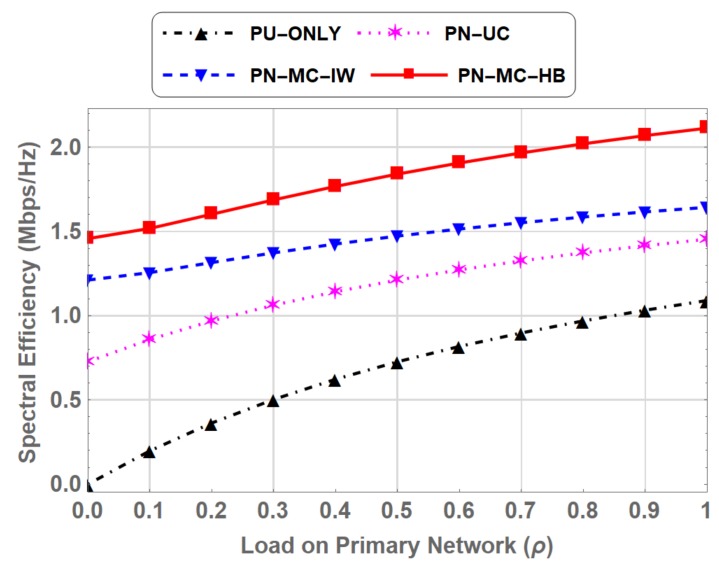
Comparison of Spectral Efficiency for PN-ONLY, PN-UC, PN-MC-IW and PN-MC-HB.

Spectrum utilization increases as the ρ increases. It is observed that spectrum utilization for PN-ONLY, where only primary network exists, is poor than the other three schemes, where primary and CRN both exist. Coexistence of the secondary network with the primary network increase the total number of users in the spectrum. Thus the spectrum utilization increase in PN-UC. Introduction of classes of SUs further increases the spectrum utilization. Spectrum utilization in PN-MC-HB is higher compared to other three throughout the entire range of ρ. The gain is also observed in spectral efficiency of the system which is the transmission rate per unit bandwidth [39]. This improvement in spectrum utilization and spectral efficiency for PN-MC-HB compared to PN-MC-IW is due to the fact that when SU is interrupted by the PU, instead of waiting on the channel to resume its transmission, switches to underlay spectrum access, hence efficiently utilizing the spectrum. At peak primary network load (ρ=1), the spectral efficiency in PN-MC-HB is 2.113 Mbps/Hz compared to 1.644 Mbps/Hz in PN-MC-IW reflecting a 28.5 % increase as shown in Figure 14.

### 5.3. Throughput

Throughput of the system is the amount of data transmitted collectively in a unit time by the primary and secondary users. As the steady state analysis gives the stationary probabilities $\pi_{\beta }$, which represent the probability of system being in state β where $\beta \in \left\{P,S,H,L\right\}$. It can equivalently be viewed as the ratio of allocation time of state β to the reference time [40]. The average throughput of the system in PN-ONLY is given by:

(29) $$T_{1}=\pi_{P}r,$$

where, *r* is the maximum achievable data rate when PU is only operating in the spectrum. For PN-UC, when spectrum is either occupied by PU or SU, the average throughput is given by:

(30) $$T_{2}=\pi_{P}r+\pi_{S}r.$$

The average throughput of the PN-MC-IW, where multi-class SUs along with primary network operating in interweave spectrum access as given below:

(31) $$T_{3}=\pi_{P}r+\pi_{H}r+\pi_{L}r.$$

**Figure 15:**
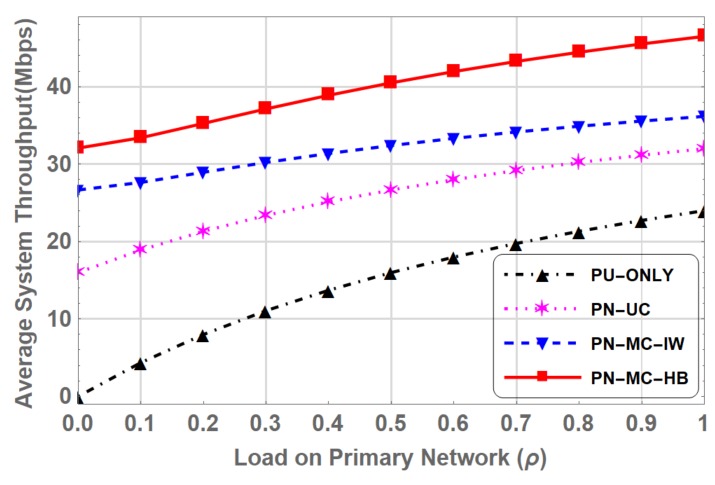
Comparison of Average System Throughput for PN-ONLY, PN-UC, PN-MC-IW and PN-MC-HB.

Finally, in PN-MC-HB where multi-class prioritized SUs operating in hybrid interweave-underlay spectrum access, the throughput of the system is given by the following relation,

(32) $$T_{4}=\pi_{P}r+\pi_{H}r+\pi_{L}r+\pi_{ph}r_{u}^{h}+\pi_{pl}r_{u}^{l}+\pi_{hl}r_{u}^{l},$$

where, *r* and $r_{u}^{\gamma }$ are defined in (1) and (2). Figure 15 shows the comparison of average throughput of the system as a function of load on primary network (ρ) for PN-ONLY, PN-UC, PN-MC-IW and PN-MC-HB. The average system throughput is highest for PN-MC-HB throughout the entire range of load on primary network (ρ).

**Figure 16:**
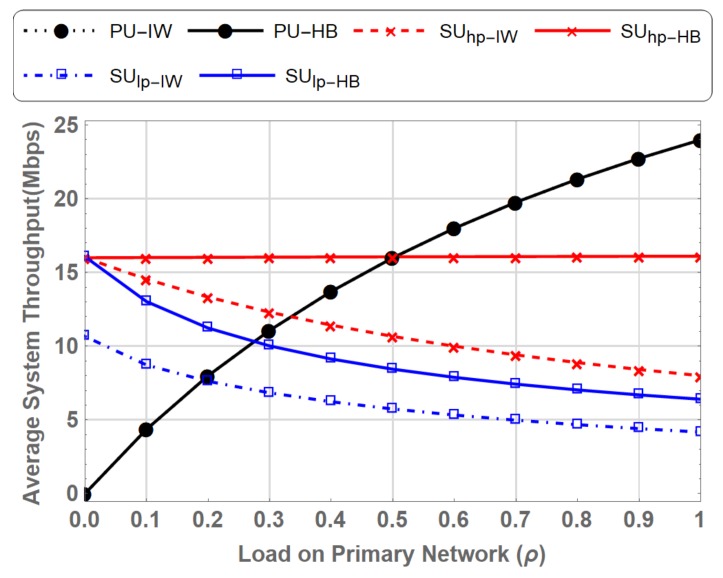
Comparison of Average System Throughput for PN-MC-IW and PN-MC-HB.

Figure 16 depicts the contributions of PU and the multi-class prioritized SUs to the average system throughput. The average data rate achieved by the $SU_{hp}$ shows a 2 fold increase at ρ=1 in case of PN-MC-HB, which increases from 8 Mbps to 16.10 Mbps compared to PN-MC-IW. It is also evident that the average throughput of the $SU_{hp}$ decreases in PN-MC-IW as the load on primary network increases. This is primarily due to the fact that very few opportunities are left for the SUs, making it difficult to meet the QoS requirements. Similarly, $SU_{lp}$ also benefits from the hybrid spectrum access, which is reflected by the increase in throughput from 4.1739 Mbps to almost 6.40 Mbps at ρ=1. This gain, though, is not as high as that for $SU_{hp}$ due to the fact that $SU_{lp}$ are preempted by the PU as well as $SU_{hp}$ during data transmission.

### 5.4. Extended Data Delivery Time

**Figure 17:**
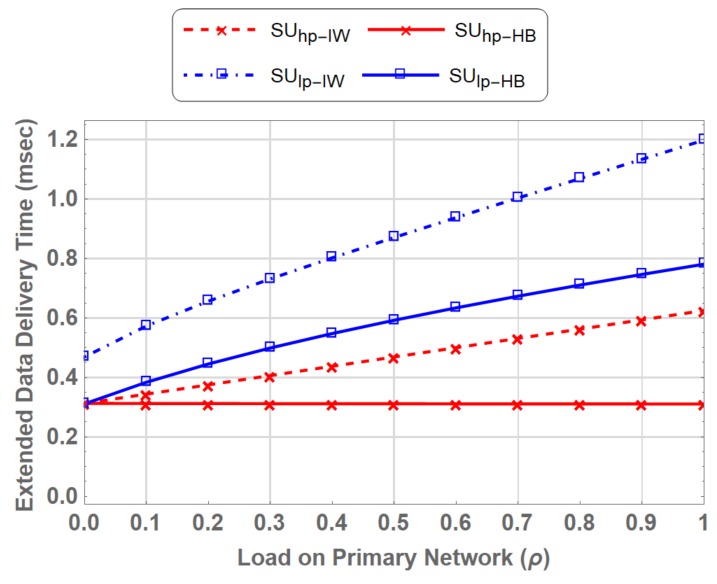
Comparison of Extended Data Delivery Time for PN-MC-IW and PN-MC-HB.

Extended data delivery time is the total time elapsed by the multi-class SUs in transmitting their complete data packets i.e., the instant SU starts its transmission until the instant it completes its data transmission. This includes the waiting time during spectrum handoff. SU may encounters multiple interruptions during its service, which increases the extended data delivery time. Figure 17 shows a comparison of EDDT for SUs in PN-MC-IW and PN-MC-HB. At ρ=0, which means when entire PU spectrum is available to SUs, the EDDT for both the interweave and hybrid spectrum access are the same at around 0.3125 ms. EDDT for the $SU_{lp-IW}$ is higher i.e., 0.468 ms, due to the fact that even at ρ=0, the priority is given to the $SU_{hp-IW}$ due to its delay constraint requirements.

When the ρ increases, the number of opportunities gradually decreases for the SUs. In the case $SU_{hp-IW}$, $\left(SU_{hp}\right)$ has to wait on the channel, which increases the EDDT, e.g., at ρ=0.9, when almost 90% of the spectrum is occupied by PU, $SU_{hp}$ is deprived of channel access for long duration, which results in fold increase in EDDT compared at ρ=0. However, in $SU_{hp-HB}$, where the interrupted user switches to underlay access mode and transmits some data rather waiting on the channel the EDDT increases only by 2.47 % from 0.3125 ms (ρ=0) to 0.32055 ms (ρ=0.9). It is evident from the Figure 17 that EDDT is reduced significantly in hybrid interweave-underlay spectrum access compared to that in the interweave only spectrum access.

**Figure 18:**
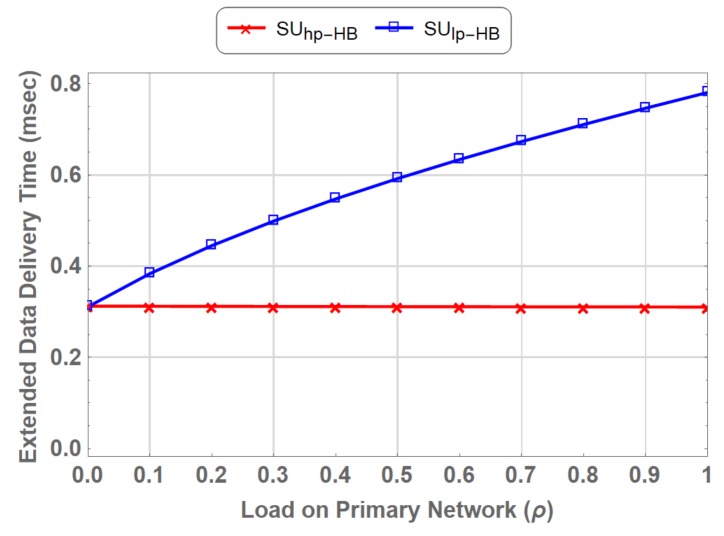
Comparison of Extended Data Delivery Time for Delay-Sensitive Traffic vs Delay-Tolerant Traffic in PN-MC-HB.

Figure 18 shows a gain in EDDT of $SU_{hp}$ over $SU_{lp}$ in PN-MC-HB. It is observed that the EDDT is reduced to 60 % in $SU_{hp-HB}$ compared to $SU_{lp-HB}$. This difference is due to the prioritized channel access and preemption policy that allows $SU_{hp}$ to preempt $SU_{lp}$.

## 6. Conclusions

The hybrid spectrum sharing schemes integrating mixed interweave-underlay access strategies in a CRN have emerged as highly promising in improving performance and thus can help meet the desired QoS requirements of SUs. This work has focused on hybrid interweave-underlay spectrum access for multi-class SUs for prioritized traffic with varied QoS targets. The spectrum handoff with hybrid interweave-underlay spectrum access scheme is modelled as a continuous time Markov chain to study the interactions between PUs and the multi-class SUs. The results indicate significant performance improvement in spectrum utilization, average system throughput and extended data delivery time for a multi-class CRN with hybrid spectrum access when compared with the performance of a conventional CRN using single homogeneous interweave only spectrum access. The overall spectral efficiency with hybrid interweave-underlay spectrum access for multi-class SUs improves by almost 28.5% compared to that for interweave only access. Similarly, significant improvement is observed in both the delay performance as well as average system throughput. The average transmission delay for the delay-sensitive SU traffic is 60 % less compared to that for delay-tolerant SU traffic. Therefore, the proposed scheme can meet the QoS requirement of the delay-sensitive SU traffic while avoiding the excessive delay caused by the frequent interruption by the high traffic PUs. The analysis framework developed as part of this work can be used to design a method for admission control in distributed CRN with multi-class SUs having different delay requirements which can further reduce the excessive delay caused by frequent spectrum handoff for delay-tolerant SUs.

## Figures and Tables

**Table 1:** Comparison of related work and proposed work in terms of Markov-based hybrid cognitive radio network (CRN) for multi-class secondary users (SU) under power constrained data rate.

	Hybrid Interweave Underlay Spectrum Access	Markov-Based Modelling and Analysis	Prioritized Multi Class SU Traffic	Consideration of Power Constrained Data Rate in Underlay Access	Throughput	Delay
Bayrakdar et al. [14]	✗	✗	✓	✗	✗	✓
Wu Yeqing et al. [15]	✗	✗	✓	✗	✗	✓
Zahed Salah et al. [16]	✗	✗	✓	✗	✗	✓
Zhang Lei et al. [17]	✗	✗	✓	✗	✗	✗
P. Thakur et al. [18]	✓	✗	✗	✓	✓	✗
A. Bhowmick et al. [19]	✓	✗	✗	✓	✓	✗
Chu Thi et al. [20,21]	✓	✓	✗	✓	✓	✗
Jie Hu et al. [22]	✓	✗	✗	✓	✗	✓
F.Cuomo et al. [23,24]	✓	✗	✗	✓	✗	✗
Wang Beibei et al. [25]	✓	✗	✗	✓	✓	✗
Hu Han et al. [26]	✓	✓	✗	✗	✗	✗
Osama Mir et al. [27]	✗	✓	✗	✗	✓	✓
A. Zahmati et al. [28]	✓	✗	✗	✗	✓	✗
El Azaly et al. [29]	✗	✓	✗	✗	✗	✗
V. Tumuluru et al. [30]	✗	✓	✓	✗	✗	✓
Proposed	✓	✓	✓	✓	✓	✓

**Table 2:** System Parameters.

System Parameters	
No. of Primary Channel ($N_{ch}$)	1
Load on primary network (ρ)	[0.0–1.0]
Mean SU arrival rate ($\lambda_{s}$)	2
Mean High Priority ($SU_{hp}$) arrival rate ($\lambda_{h}$)	1
Mean Low Priority ($SU_{lp}$) arrival rate ($\lambda_{l}$)	1
Mean SU Departure rate ($\mu_{s}$)	4
Mean High Priority ($SU_{hp}$) Departure rate ($\mu_{h}$)	2
Mean Low Priority ($SU_{lp}$) Departure rate ($\mu_{l}$)	2
Mean High Priority ($SU_{hp}$) Departure rate - underlay ($\mu_{hu}$)	0.01
Mean Low Priority ($SU_{lp}$) Departure rate - underlay ($\mu_{lu}$)	0.01
File Size (Size)	5 Kbit
Transmission rate (IEEE 802.11a) - Interweave ($R_{i}$)	48 Mbps
Transmission rate (IEEE 802.11a) - Underlay ($R_{u}$)	18 Mbps
Channel Bandwidth (IEEE 802.11a) (W)	22 MHz

## Abbreviations

The following abbreviations are used in this manuscript:

AWGN	Additive White Gaussian Noise
CRN	Cognitive Radio Network
CTMC	Continuous Time Markov Chain
EDDT	Extended Data Delivery Time
HMM	Hidden Markov Model
MDP	Markov Decision Process
NPRP	Non-Preemptive Resume Priority
PRP	Preemptive Resume Priority
PU	Primary User
QoS	Quality of Service
SU	Secondary User
SINR	Signal to Interference plus Noise Ratio
VoIP	Voice over Internet Protocol
